# Evaluation of soluble suppression of tumorigenicity 2 (sST2) as serum marker for liver fibrosis

**DOI:** 10.1186/s12876-023-03116-4

**Published:** 2024-01-30

**Authors:** Florian F. Hildenbrand, Barbara Illi, Stefanie von Felten, Jacqueline Bachofner, Joanna Gawinecka, Arnold von Eckardstein, Beat Müllhaupt, Joachim C. Mertens, Sena Blümel

**Affiliations:** 1 Division of Gastroenterology and Hepatology, University Hospital Zurich, University of Zurich, Rämistrasse 100, 8091 Zurich, Switzerland; 2 Division of Gastroenterology and Hepatology, Stadtspital Zurich, Zurich, Switzerland; 3 Department of Biostatistics, Epidemiology, Biostatistics and Prevention Institute, University of Zurich, Zurich, Switzerland; 4 Institute of Clinical Chemistry, University Hospital Zurich, University of Zurich, Zurich, Switzerland; 5 Center of Gastroenterology, Klinik Hirslanden, Zurich, Switzerland

**Keywords:** sST2, CK-18, HCV, Liver fibrosis, Elastography, FIB-4

## Abstract

**Background & aims:**

With the increase in patients at risk of advanced liver disease due to the obesity epidemic, there will be a need for simple screening tools for advanced liver fibrosis. Soluble suppression of tumorigenicity 2 (sST2) is a serum biomarker for fibrotic processes. The aim of this study was to evaluate sST2 as marker for liver fibrosis in patients successfully treated for chronic hepatitis C.

**Methods:**

424 patients from the Swiss Hepatitis C Cohort Study were screened for inclusion in this post-hoc cohort study. Inclusion criteria were sustained virological response (SVR), available elastography (VCTE) and serum samples for biomarker analysis before and after treatment. For the validation of sST2, values were compared to VCTE, FIB-4 and APRI using Spearman’s correlation and AUROC analyses.

**Results:**

Data of 164 subjects were finally analyzed. Median sST2 values slightly increased with VCTE-derived fibrosis stages and remained stable after reaching SVR within the respective fibrosis stage, suggesting that sST2 is not influenced by liver inflammation. However, correlation of sST2 pre- and post-treatment with VCTE was fair (Spearman’s rho = 0.39 and rho = 0.36). The area under the curve (AUROC) for sST2 in detecting VCTE-defined F4 fibrosis (vs. F0-F3) before therapy was 0.74 (95%CI 0.65–0.83), and 0.67(95%CI 0.56–0.78) for the discrimination of F3/F4 fibrosis vs. F0-F2. Adding sST2 to either APRI or FIB-4, respectively, increased diagnostic performance of both tests.

**Conclusions:**

sST2 can potentially identify patients with advanced fibrosis as a single serum marker and in combination with APRI and FIB-4.

## Introduction

In chronic liver disease, hepatocyte injury and inflammation lead to a progressive fibrotic remodelling of the liver tissue which ultimately ends in liver cirrhosis, a condition that is associated with high morbidity and mortality [1, 2]. Unlike earlier stages of liver fibrosis, cirrhosis is barely reversible, even if the underlying liver disease is treated [3, 4]. With the obesity epidemic, which is accompanied by increasing numbers of patients with metabolic-associated steatotic liver disease (MASLD), it is therefore crucial to identify patients with progressive liver fibrosis as early as possible.

Liver biopsy remains the gold standard for assessing the degree of fibrosis and the presence of cirrhosis. Recent studies have raised concerns about its status as a gold standard, as there is relevant inter-observer variability and variability in histological results, implying that even a perfect biomarker cannot achieve an AUROC value of > 0.90 [5]. In addition, it bears the risk of bleeding complications and is costly, especially when performed repeatedly. Therefore, non-invasive techniques have been validated to evaluate liver fibrosis [5–7]. Vibration-controlled transient elastography (VCTE) easily assesses liver fibrosis, but requires appropriate equipment and expertise [8–11]. Serum-based scores, such as Fibrosis-4 Score (FIB-4) and aspartate aminotransferase-platelet ratio index (APRI) show acceptable sensitivity and specificity particularly to exclude advanced fibrosis and cirrhosis in chronic liver disease [5, 12, 13]. They are associated with a negligible risk, readily available through routine laboratory and do not require special equipment. However, using a single biomarker might be even more convenient, especially for primary care physicians, to screen for advanced liver fibrosis.

The soluble isoform of Suppression of Tumorigenicity 2 (sST2) belongs to the interleukin (IL)-1 receptor superfamily. As a biomarker, sST2 might have the potential to be used as screening parameter for progressive liver disease [14–18], because it is thought to be a surrogate for fibrotic processes [12, 19–21]. In normal conditions, the serum concentration of sST2 is below the detectable level. Elevated levels of sST2 have been reported in patients with autoimmune diseases, lung disease and heart failure [16, 22, 23]. In human fibrotic liver disease, the IL-33/ST2 signalling pathway is upregulated [24], it induces hepatic stellate cell activation and as a consequence facilitates progression to liver fibrosis [20, 21, 24, 25]. sST2, which is measurable in serum, correlates well with the hepatic IL-33/ST2 activation in liver fibrosis [12, 21, 26].

A biomarker that has already been studied in liver disease is cytokeratine 18 (CK-18). CK-18 is a major intermediate filament protein in hepatocytes [27]. CK-18 levels are elevated in the presence of necrosis and apoptosis in liver disease [28] with high levels of CK-18 being present in patients with non-alcoholic fatty liver disease, non-alcoholic steatohepatitis and chronic hepatitis C (CHC) [27, 28].

The aim of this study was to evaluate sST2 as serum marker for liver fibrosis. To this end, we assessed serum level of sST2 alone and in combination with CK-18, FIB-4 and APRI in patients treated for CHC with treatment-induced regression of fibrosis as detected by VCTE [13].

## Methods

### Study design and study population

This study was designed and conducted as a post-hoc single centre cohort study at the Department of Gastroenterology and Hepatology, at the University Hospital Zurich, Switzerland. Data were collected within the Swiss Hepatitis C Cohort Study (SCCS) [13, 29]. Data of patients treated for CHC between March 2014 und December 2015 and available serum samples for this investigation were included in the analysis.

This study is a sub-group analysis of a study published by Bachofner et al. [13, 29] with available serum samples for the post-hoc analysis of CK-18 and sST2 either before or after treatment or both. Inclusion criteria were a direct acting agent-based (DAA) therapy for CHC, available data on treatment outcome, corresponding VCTE values as well as available lab values for the calculation of APRI and FIB-4 scores before and/or after DAA therapy. Sustained virological response (SVR) was defined as undetectable HCV RNA 12 weeks after end of treatment; if HCV RNA was still detectable, this was defined as non-SVR according to current guidelines [5, 6]. Plasma samples for determination of sST2 and CK-18 levels were collected at the time of liver stiffness evaluation before and after treatment. Depending on cirrhosis stage, genotype, and treatment history, therapy was administered for 8–24 weeks with or without ribavirin according to international guidelines at the time the study was conducted. Based on the DAA therapies available in Switzerland in 2014 and 2015 the most frequently used DAA therapy was Sofosbuvir/Ledipasvir (SOF/LED).

Liver stiffness was assessed by VCTE (FibroScan™, Echosens, Paris, France). Patients underwent measurements within 3 months prior as well as 12 weeks after the HCV treatment. The degree of fibrosis was derived from VCTE values using HCV-specific cut-off values according to EASL guidelines (F0: VCTE < 5.1 kPa; F1: VCTE ≥ 5.1 kPa; F2: VCTE > 8.4 kPa; F3: VCTE > 9.6 kPa; F4: VCTE > 12.8 kPa) [5, 6]. Only VCTE measurements after a six-hour fasting interval were included in which the interquartile range was less than 30% of the median value (IQR/med < 30%) and more than 60% valid measurements were available. After recommendation by the device software, the XL probe was used for obese patients [30]. APRI was calculated with the formula: [AST (IU/l)/AST (Upper Limit of Normal-IU/l)/Platelet count (109/l) × 100]; APRI < 1.0 rules out advanced fibrosis and APRI > 2.0 predicts advanced fibrosis. FIB-4 was determined according to the formula: [Age (years) × AST level (IU/l)]/[(Platelet count (10^9^/l) × ALT (IU/l)]; FIB-4 < 1.3 rules out advanced fibrosis and FIB-4 > 3.25 predicts advanced fibrosis [5, 6, 31, 32].

### Biological sample handling and processing

All serum samples of the SCCS were stored at −80 degrees Celsius until analyses were performed. Samples were analysed by the Department of Clinical Chemistry, University Hospital Zurich. sST2 was measured using a validated ELISA (Presage™, Ruwag Diagnostics, Bettlach, Switzerland) according to the manufacturer’s manual with a standard curve spanning the range of 3.1 to 200.0 ng/ml [33, 34]. CK-18 was measured using a validated ELISA (M30-Apoptosense Previva™ 10,011, TECOmedical AG, Sissach, Switzerland) according to the manufacturer’s manual.

All samples were measured in duplicates. The mean value from both measurements was used for analysis.

### Ethics

All patients provided written informed consent for the inclusion into SCCS (KEK ZH number EK-695). The study protocol for the presented study was in accordance with the ethical guidelines of the Declaration of Helsinki and was approved by the ethics committee of the Canton of Zurich (BASEC number 2016-00341).

### Statistical methods

All analyses were performed using the R system for statistical computing and graphics (R Core Team (2022), Vienna, Austria).

Spearman’s correlation coefficients were calculated for assessing the association of VCTE measurements with sST2 and CK-18, as well as with APRI and FIB-4. The biomarkers sST2, CK-18 and the established scores APRI, and FIB-4 were used as explanatory variables in logistic regression models on fibrosis degree (both dichotomized version) before DAA treatment. First, each explanatory variable was used alone. Then, sST2 and CK-18 were combined with APRI (APRI + sST2 + CK-18; APRI + sST2; APRI + CK-18) or with FIB-4 (FIB-4 + sST2 + CK-18; FIB-4 + sST2; FIB-4 + CK-18). Receiver operating characteristic curves (ROC) were drawn for all models to compare the diagnostic ability of biomarkers with APRI and FIB-4 and combinations of the biomarkers with APRI and FIB4, respectively. Area under the receiver operating curve (AUROC) was estimated with a 95% confidence interval.

## Results

### Study population and patient characteristics

**Fig. 1:**
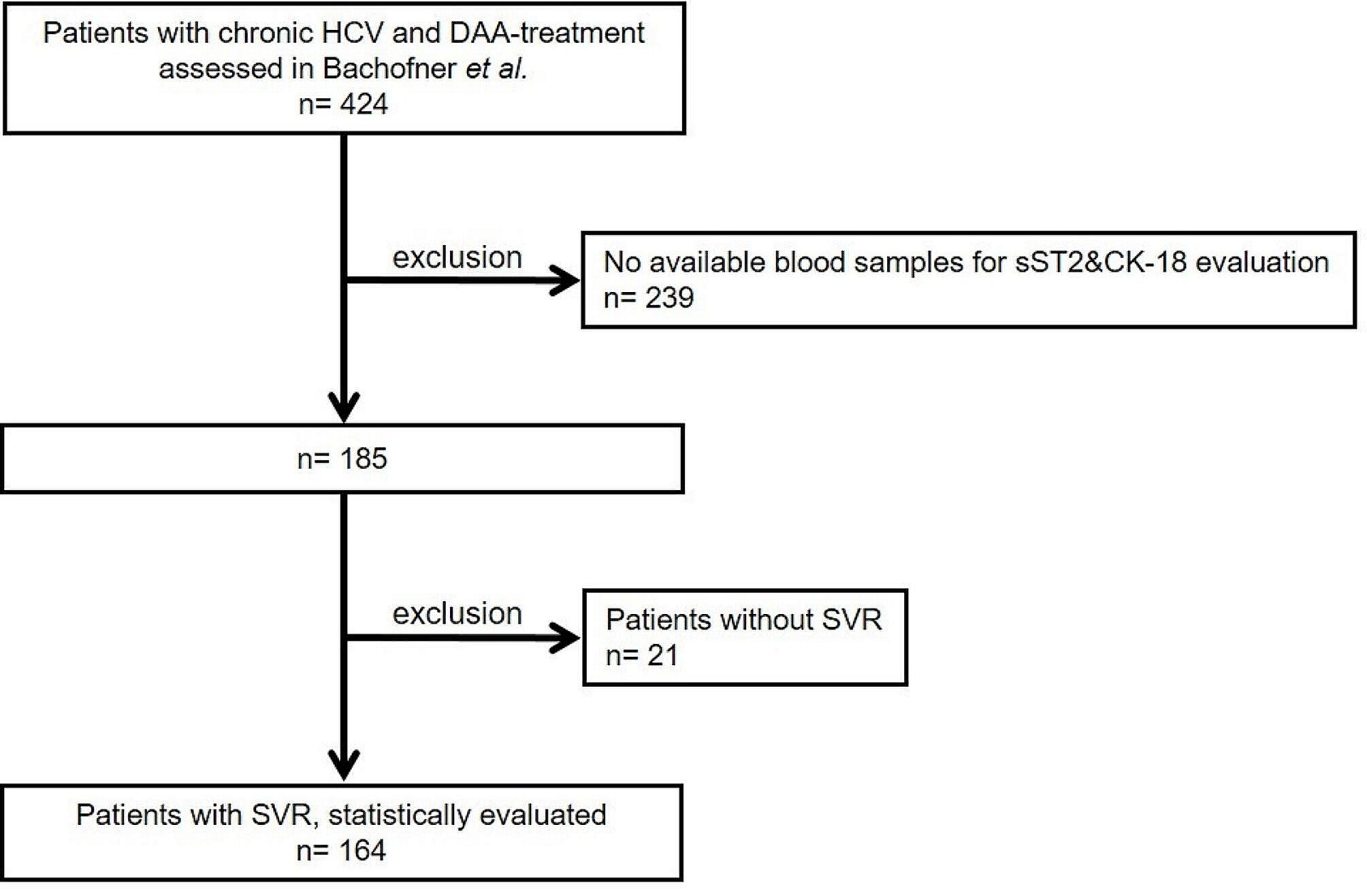
**Study flow chart.** DAA: Direct acting anti-viral; HCV: Hepatitis C infection; SVR: Sustained virological response

Of the 424 patients included in the study of Bachofner et al. [13], 239 were excluded due to missing blood samples. Additionally, 21 patients without SVR were excluded. Finally, 164 patients treated with DAA for CHC with SVR and evaluable blood samples, were included (ref. Fig. 1). Patient characteristics of the investigated 164 patients with SVR are given in Table 1.

**Table 1:** **Patient characteristics:** mean and standard deviation [SD] are given for continuous variables with approximate normal distribution, median and inter-quartile range [IQR] for skewed continuous variables and frequencies [percentages, %] for categorical variables

	*N* = 164	% missing values
Age, years (mean ± SD)*	56.5 [± 10.2]	0
Male sex (n, [%])	102 [62.2]	0
BMI, kg/m^2^ (mean, [± SD])	25.8 [± 4.6]	24.9
Viral Load, IU/ml (median [IQR])	15’000’000 [607’500, 31’000’000]	1.2
HCV Genotype (n, [%])		0.6
–1	111[8.1]	
–2	8 [4.9]	
–3	23 [14.1]	
–4	20 [12.3]	
–6	1 [0.6]	
Coinfection (n, [%])		0.6
–Hepatitis B	2 [1.2]	
–HIV	3 [1.8]	
Concomitant ALD (n, [%])	30 [18.4]	0.6
Concomitant MASLD (n, [%])	2 [1.2]	0.6

*Abbreviations*: ALD = Alcoholic liver disease, BMI = Body mass index, DAA = Direct acting agent, HCV = Hepatitis C Virus, MASLD = metabolic dysfunction associated steatotic liver disease

The predominant genotype in this European cohort was genotype 1. Co-existing liver disease, such as co-infections with hepatitis B or HIV, alcoholic liver disease and MASLD, was present in 23.9% of patients. The majority of patients had a high fibrosis degree (F3 or F4). The rather low body mass index and the low proportion of MASLD was remarkable.

Details of treatment-related changes in the assessed parameters are given in Table 2.

**Table 2:** **Serum and VCTE values before and after treatment.** Mean and standard deviation [SD] are given for continuous variables with approximate normal distribution, median and inter-quartile range [IQR] for skewed continuous variables and frequencies [percentages, %] for categorical variables

	before DAA	after DAA	Missing values^§^ (%)	p-value
n	164	164		
sST2, ng/ml (median [IQR])	32.8 [22.8, 43.1]	27.5 [20.0, 36.8]	19.2	*p* < 0,001
CK-18, U/l (median [IQR])	178.3 [87.0, 370.4]	52 [29.9, 83.0]	18.9	*p* < 0,001
VCTE, kPa (median [IQR])	12.6 [8.8, 18.5]	7.9 [6.1, 13.0]	2.1	*p* < 0,00
Fibrosis grade (n [%])^#^			2.1	1
–F0	10 [6.1]	24 [15.3]		*p* < 0,001
–F1	30 [18.3]	60 [38.2]		
–F2	8 [4.9]	7 [4.5]		
–F3	38 [23.2]	26 [16.6]		
–F4	78 [47.6]	40 [25.5]		
FIB-4 (median [IQR])	2.6 [1.7, 4.7]	1.8 [1.3, 2.7]	11.0	*p* < 0,001
APRI (median [IQR])	1.1 [0.6, 2.0]	0.4 [0.3, 0.6]	11.0	*p* < 0,001
AST, U/l (median [IQR])	65.0 [45.3, 101.8]	26.0 [22.0, 33.0]	4.6	*p* < 0,001
ALT, U/l (median [IQR])	79.0 [51.0, 128.0]	22.0 [16.0, 32.0]	3.4	*p* < 0,001
Bilirubin, mmol/l (median [IQR])	11.0 [8.0, 16.0]	9.0 [7.0, 15.0]	4.0	*p* < 0,001
γ-GT, IU/l (median [IQR])	83.0 [50.0, 184.0]	33.0 [19.8, 55.3]	8.8	*p* < 0,001

#Categories were derived from VCTE measurements according to EASL Guidelines [5]. § gives the percentage of total missing values, i.e., before and after DAA in 328 total possible measurements. *Abbreviations*: ALT = Alanine amino transferase, APRI = Aspartate amino transferase to Platelet Ratio Index, AST = Aspartate amino transferase, CK-18 = Cytokeratine 18, FIB-4 = Fibrosis-4 score, γ-GT = Gamma Glutamyltransferase, sST2 = soluble Suppression of tumorigenicity 2, VCTE = Vibration-controlled transient elastography

Successful treatment led to a normalization of serum transaminases. Similarly, there was a marked decrease in CK-18 and, to a much lesser extent, also for sST2. After successful treatment, VCTE values decreased from a median value in the range of F3 to a median value in the range of F1, likewise, median APRI values dropped from the grey zone (F2/F3) to values in the range of F1/F0 (i.e., to values below 0.5). Thus, APRI matched the values of VCTE. Median FIB-4 values remained in the grey zone for liver fibrosis (i.e., above 1.45 and below 3.25.

### sST2 as potential marker for liver fibrosis

**Fig. 2:**
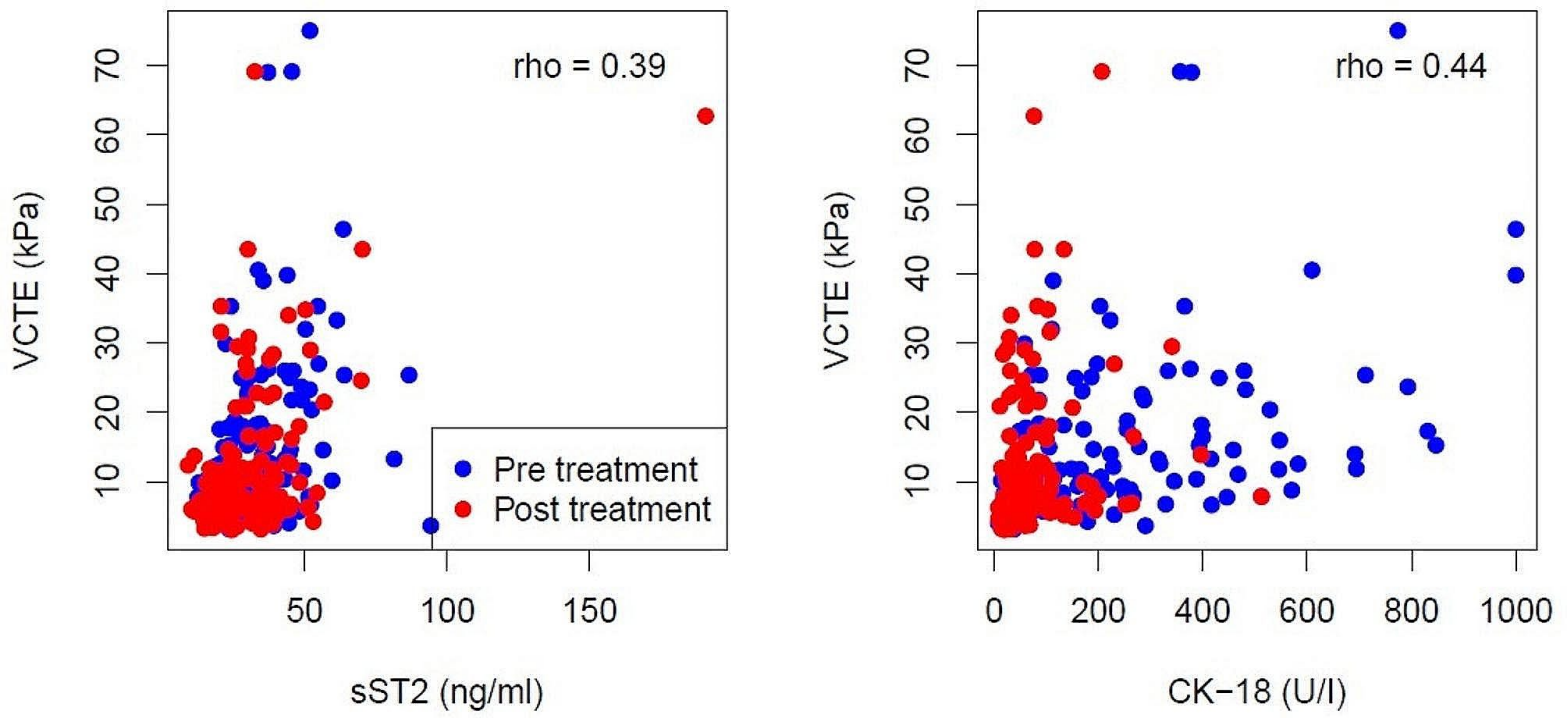
**Bivariate scatterplots of actual measurements between VCTE and sST2 and VCTE and CK-18.** Combined presentation of pre- and post-treatment measurements (blue and red circles, respectively). The spearman correlation coefficient rho for the combined data is shown in the panels. Rho pre- and post-treatment for VCTE and sST2 were 0.39 and 0.36, respectively. Rho pre- and post-treatment for VCTE and CK-18 were 0.44 and 0.30, respectively

To understand, if sST2 is influenced by liver inflammation, and if it might serve as a marker for liver fibrosis, values of sST2 and CK-18 (which is a marker for apoptosis and inflammation) were correlated with VCTE values (Fig. 2). While the correlation of CK-18 and VCTE became slightly worse after successful HCV elimination, the correlation of sST2 and VCTE remained rather stable. This suggests that sST2 is influenced to a comparable extent by inflammation than VCTE.

**Fig. 3:**
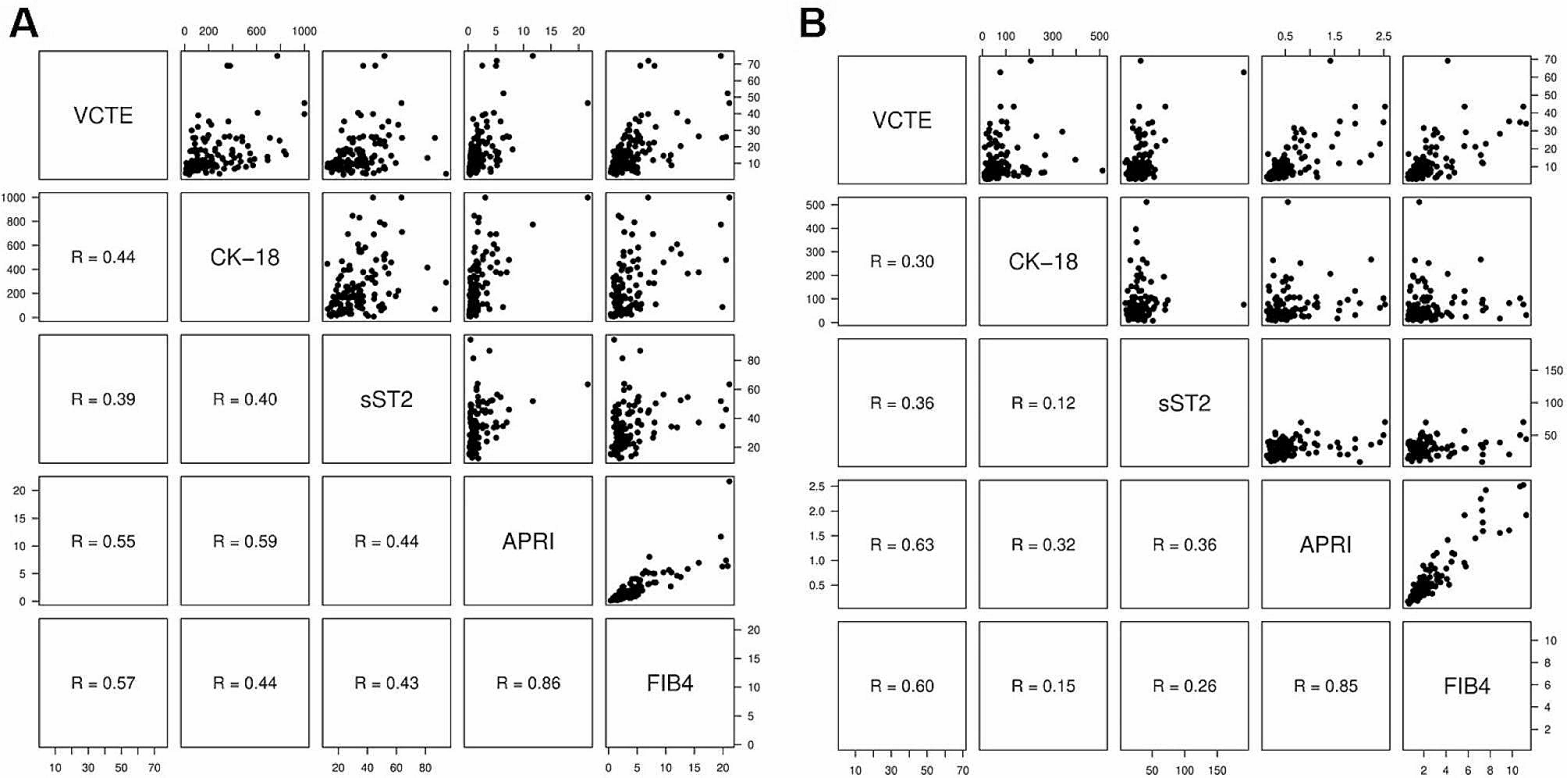
**Correlations of values for VCTE, CK-18, sST2, APRI and FIB-4**. Scatter plots with spearman correlation coefficients for values before treatment (panel A) and after treatment (panel B)

To characterize our cohort also for established fibrosis scores, Fig. 3 shows the interrelation of values for sST2, CK-18, VCTE, APRI and FIB-4 for measurements before and after treatment. While APRI and FIB-4 strongly correlated with each other before and after treatment, their correlation with VCTE values was clearly weaker. The correlation of sST2 with both APRI and FIB-4 was in the range of the correlation of sST2 and VCTE (as also shown in Fig. 2). While the correlation of sST2 and APRI remained about stable after treatment, the correlation of sST2 with FIB-4 became worse. Correlation of sST2 and CK-18 was fair before treatment and became even worse after treatment.

**Fig. 4:**
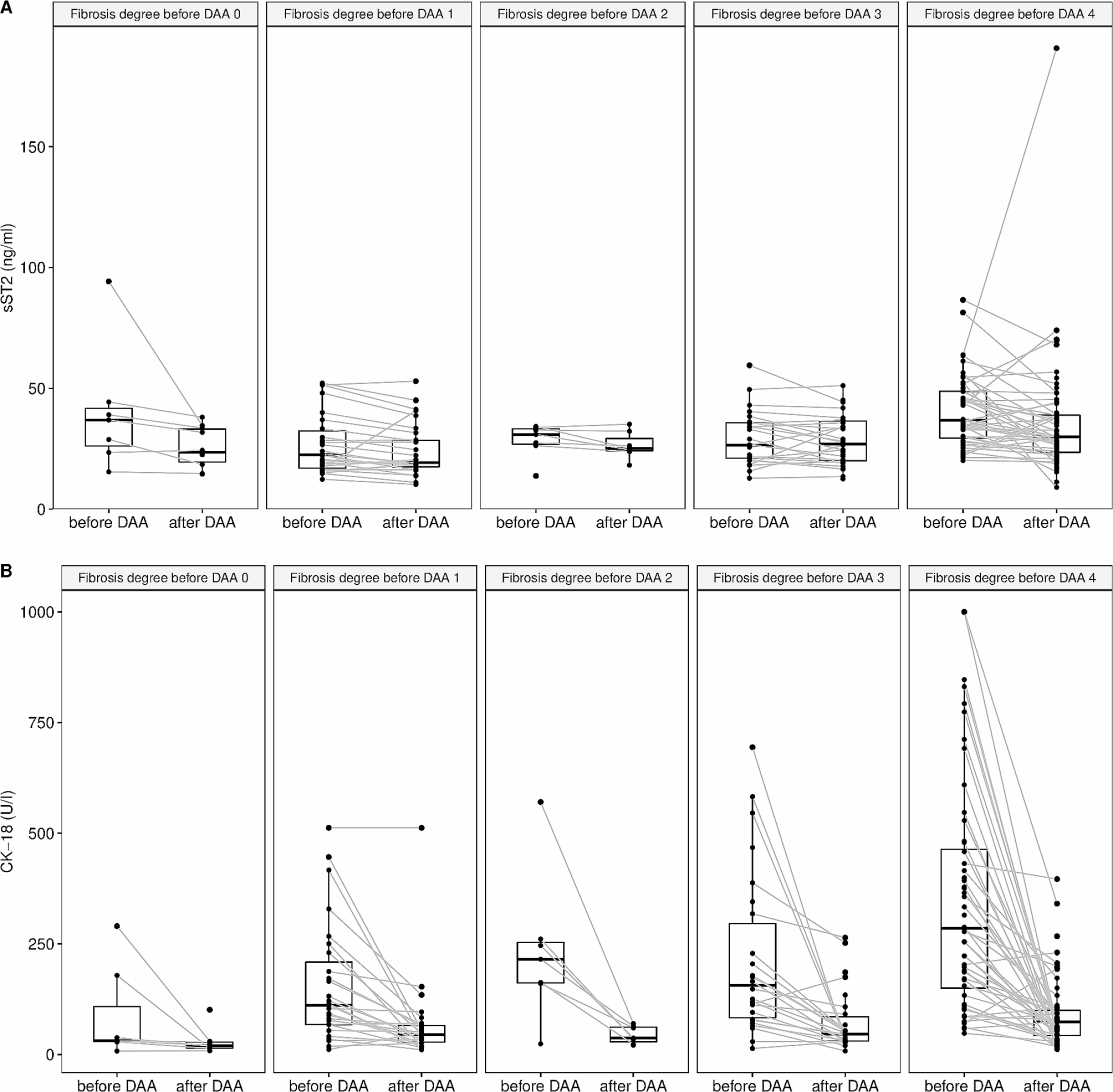
**Line plots for sST2 (panel A) and CK-18 (panel B) according to VCTE-derived fibrosis stages before treatment with DAA.** Dots display individual measurements and lines connect the paired measurements of individual patients. Boxes indicate the median and interquartile range. DAA: direct acting anti-viral

To further clarify the interrelation of VCTE-derived liver fibrosis stage and sST2 level, pre- and post-treatment sST2 values were analysed according to fibrosis grade (ref Figure 4A). Median sST2 values slightly rose with increasing fibrosis stage and treatment did not affect median sST2 values. In contrast, CK-18, which markedly increased before treatment, decreased to low levels over all fibrosis stages after treatment (ref. Fig. 4B).

### Applicability of sST2 for the prediction of liver fibrosis

To check for the ability of sST2 to predict advanced liver fibrosis (i.e., F3/F4 fibrosis), we derived ROC curves with area under the ROC curve (AUROC) for APRI, FIB-4, CK-18 and sST2 from values before treatment in patients with SVR.

**Fig. 5:**
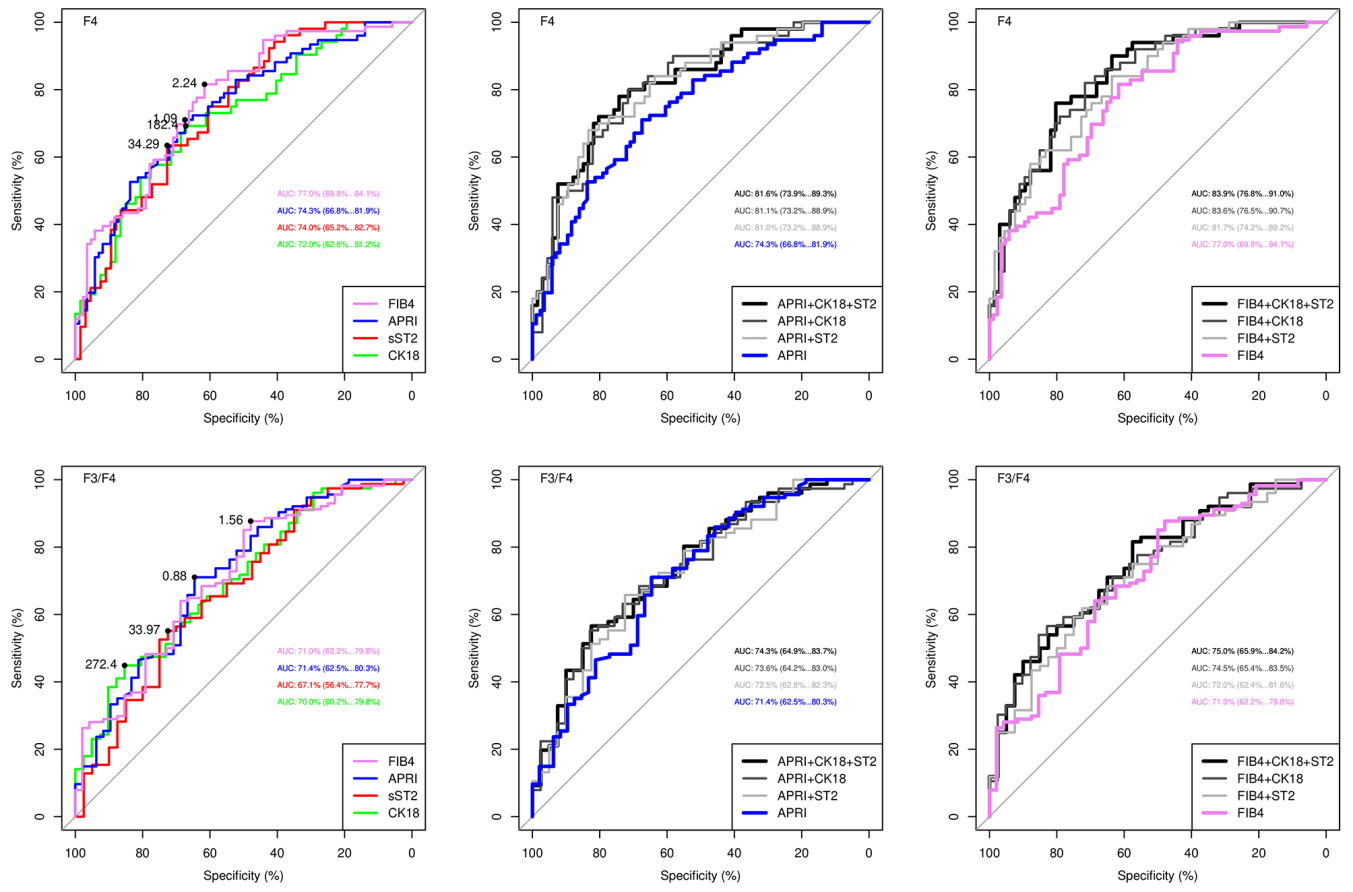
**Comparison of ROC curves for APRI, FIB-4, CK-18 and sST2 before DAA treatment.** ROC curves for single explanatory variables and combined models. Best cut-off values were identified using youden’s index and are shown as closed black circles in the panels. **Upper panels**: Distinction of F4 fibrosis from F0-F3 fibrosis. **Lower panels**: Distinction of F3/4 fibrosis from F0-F2 fibrosis

Figure 5 shows the performance of the different parameters in our cohort to distinguish F4 from lower fibrosis stages F0-F3 (upper panels) or F3/F4 fibrosis from F0-F2 fibrosis (lower panels).

Single explanatory variables all show comparable AUROC, but AUROC of APRI and FIB-4 were slightly higher than the AUROC of sST2 and CK-18, respectively. However, adding sST2 or CK-18 to APRI or FIB-4, respectively, slightly increased the AUROC for detecting F4 fibrosis before treatment. The same was true for the distinction of F3/4 vs. F0-F2.

Using an sST2 cut-off value of 34.29 ng/ml best distinguished F4 and F0-F3 fibrosis. However, due to the low AUROC, sensitivity and specificity were rather low (< 70%), and even lower for the discrimination of F3/4 and F0-F2 fibrosis (using an sST2 value of 33.97 ng/ml, ref Figure 5). From all fibrosis marker, performance of FIB-4 was best, and the cut-off value of 1.56 had a sensitivity of > 80% (but a low specificity) for the discrimination of F3/4 from lower fibrosis stages. The performance of APRI was also slightly better than sST2, with a cut-off value of 0.88 showing a sensitivity and specificity of about 70% to distinguish F3/4 from F0-F2.

## Discussion

This study evaluates the suitability of sST2 as a marker for liver fibrosis in a Swiss cohort of patients with CHC successfully treated with DAA therapy.

Taken together, sST2 measurements are barely susceptible to inflammation-related interference and reflect well the fibrosis that is present. However, sST2 has a fair correlation with established fibrosis scores APRI and FIB-4 as well as VCTE. Nonetheless, in our cohort, it slightly improved their performance in predicting the presence of advanced liver fibrosis stages before DAA treatment.

Based on the physiological role of the IL-33/ST2 axis, sST2 is of particular interest in the assessment of liver fibrosis. It is thought that the development of fibrosis is a result of an imbalance between inflammation and anti-inflammatory or regenerative processes, which lead to the remodelling of the parenchyma [21]. sST2 has been investigated in patients with lung fibrosis and heart failure [21–23] and has been proposed as a fibrosis marker also in liver disease [26, 35]. In the human liver, tissue ST2 mRNA levels increased with increasing fibrosis stages [24], and these mRNA levels correlated well with sST2 that can be measured in plasma [12, 21, 26]. In line with these findings, we detected increased levels of sST2 with increasing fibrosis stages. This finding was supported by the correlation of sST2 with VCTE values (*r* = 0.39). However, correlations of VCTE with APRI and FIB-4, respectively, were slightly stronger (ref. Fig. 3).

To be able to discriminate between an sST2 elevation caused by fibrosis or inflammation/apoptosis, respectively, we additionally assessed CK-18 serum values. CK-18 serum level increase in the presence of necrosis and apoptosis in liver disease [18, 29, 36–38]. High levels of CK-18 are present in patients with non-alcoholic fatty liver disease, non-alcoholic steatohepatitis and CHC [27, 28]. In our study, we could confirm those previous findings by several observations: (1) CK-18 levels clearly decreased after successful DAA treatment across all fibrosis stages (ref. Figs. 2 and 4B); (2) CK-18 decreased even in patients with cirrhosis (ref. Fig. 4B); and (3) the correlation of CK-18 with VCTE values strongly decreased after treatment (ref. Figs. 3 and 4B). Although it has been claimed that VCTE before HCV therapy not only reflects the degree of fibrosis but also the inflammatory activity and necrosis [5, 8, 10, 13, 39], VCTE was classified as adequate for the measurement of fibrosis in patients with HCV in previous studies and guidelines [5, 6]. In contrast to the findings for CK-18, sST2 level remained more stable in patients with cirrhosis who had HCV elimination (ref. Fig. 4).

The identification of patients with F3 or F4 fibrosis is clinically relevant, not only in patients with CHC [6, 7], but also in the light of the obesity epidemic resulting in increasing numbers of patients with MASLD and metabolic dysfunction associated steatohepatitis. Because liver biopsy is currently the gold standard for detecting liver fibrosis, there is a significant need for non-invasive methods [8, 39, 40]. A simple screening tool, i.e., a single biomarker, for general practitioners is urgently needed to identify patients at risk of high-grade fibrosis. To assess the diagnostic value of sST2 and to identify a cut-off value of sST2 that could discriminate between mild (F0-F2) and severe (F3/4) fibrosis (or at least discriminate cirrhosis (F4) and lower fibrosis stages (F0-F3)), we performed a ROC analysis. In this analysis, sST2 performed well as a single marker with an AUROC of 0.772 using a cut-off value of 34.2 ng/ml (ref. Fig. 5). This is in line with another study that investigated sST2 for the prediction of liver fibrosis in patients suffering mainly from hepatitis B [19]. In the study by *Moon et al.*, AUROC was 0.719 to detect a fibrosis stage of F2 or higher (cut-off value 39.9 ng/ml) and 0.772 to detect a fibrosis stage of F3 or higher (cut-off value 40.8 ng/ml), respectively.

The gold standard for detection and grading of liver fibrosis remains the liver biopsy and histological assessment [5]. In recent years, the value of this “gold standard” has been doubted [5, 41, 42], as the interpretation of the liver biopsy is hampered by inter-observer variability, variability of histological results depending on the location of biopsy, technical aspects and complications. Therefore, liver biopsy is not the ideal gold standard for biomarker evaluation, and it has been shown that an AUROC > 0.90 cannot be obtained even for a perfect biomarker [5].

Non-invasive assessment of liver fibrosis in patients with hepatitis C infection can be carried out in two ways: There are so-called physical tests, in particular VCTE and MR elastography, as well as a large number of biological methods [43, 44]. These include direct biomarkers such as hyaluronic acid (HA) and type IV collagen 7s (COL4-7 S), indirect biomarkers such as AST, Bilirubin or ALT, and commercially available tests that combine several parameters, such as FibroTest, Fibrometer™ or Hepascore [45]. Due to the constantly increasing number of biomarkers and tests available, we chose to compare sST2 with APRI and FIB-4, as recommended by the AASLD and EASL guidelines [6, 46]. 

One well-studied marker is hyaluronic acid. This has been investigated in several studies in patients with hepatitis C [47]. HA is a glycosaminoglycan polymer and a component of the extracellular matrix, especially of hepatic stellate cells. HA is an established direct biomarker of fibrosis and elevated levels are found in patients with CHC [43, 44, 47]. The assessment of HA level for the diagnosis of fibrosis is interfered by the patient’s age, a missing fasting interval and also systemic inflammation, as HA is also an acute phase protein. HA serum levels have been strongly associated with advanced stages of liver fibrosis, with AUROC for significant fibrosis ranging from 0.73 to 0.86 before therapy [47]. A meta-analysis of Egyptian studies showed that the determination of threshold values also differs markedly depending on the population studied. HA might [46] therefore be difficult to apply in daily practice [48]. It is even more difficult to categorise the results for HA after successful DAA therapy. In a recent large study by *Patel et al.*, HA demonstrated sufficient performance in the exclusion of various stages of fibrosis. In addition, HA levels correlated with a decrease in the histological activity index, but not with the change in fibrosis stage six months after end of DAA therapy [43]. This suggests that the observed change in HA levels is rather due to the decrease in liver inflammation than due to the decrease in liver fibrosis. This would also explain why in a study by *Martinez et al.*, there was a rapid rebound of HA in the absence of a response to therapy which cannot be explained by the immediate return of fibrosis [49].

To distinguish between inflammation and fibrosis, we concomitantly measured CK-18 as an inflammatory marker. Moreover, to dissect between inflammation and fibrosis, we did a subgroup analysis in patients with VCTE-derived F4 fibrosis that remained in the F4 group after DAA therapy (data not shown). In this group, sST2 remained stable, whereas CK-18 dramatically decreased. So while CK-18 somehow behaved like HA, sST2 might be more valuable to grade liver fibrosis.

Another single biomarker for the assessment of liver fibrosis is type IV collagen 7s (COL4-7-S). It is associated with an increase in basement membrane hyperplasia, which in turn is associated with an increase in liver fibrosis. Elevated COL4‐7-S levels have also been observed in other diseases such as kidney disease or pulmonary fibrosis [50]. In a retrospective study, it was shown that COL4‐7-S has an AUROC of 0.85 compared to VCTE with regard to the detection of cirrhosis. However, the best result was achieved when COL4-7 S was analysed in combination with other biomarkers [51]. A recently published study by Yamataka et al. [52] showed that a persistently elevated COL4‐7-S level before, during and after DAA therapy correlates with all-cause mortality after SVR. Histological assessment for reversion of liver fibrosis and data of serial evaluation for transient elastography after HCV eradication were not available in this study, so that an influence by inflammatory factors cannot be completely ruled out. Nevertheless, it could be shown that there is a correlation of a more fibrosis-specific marker with a relevant clinical endpoint. A comparison with our data is difficult due to the different endpoints. But this study emphasises that markers that appear to be less susceptible to inflammatory confounders may have a benefit in grading patients with CHC before treatment and at follow-up.

The possibility to investigate the behavior of sST2 in patients with liver disease in a state of pronounced inflammation and in a state of reduced or absent inflammation (i.e., before and after treatment of HCV) is a unique strength of our study. Through concomitant measurement of CK-18, we consider sST2 to be only minimally influenced by liver inflammation, making it a promising marker for the assessment of liver fibrosis. This confirms the existing assumptions of previous studies in liver diseases [1, 19, 53].

We have chosen the non-invasive assessment of fibrosis degree using FIB-4, APRI and VCTE, as this is the standard in our centre. Many comparable studies have used this method [11, 40, 45, 49, 51]. However, it must be noted that this is arbitrary and as there are numerous available serum derived non-invasive tests for liver fibrosis, our study might have yielded different results when comparing sST2 to any other of these tests. In addition, it is a limitation of our study that fibrosis degree was not derived from histology.

In conclusion, sST2 has the potential to identify patients with advanced fibrosis. As our study derived fibrosis stages from values of VCTE in patients with CHC, further studies are needed to evaluate, if sST2 can also detect liver fibrosis in other chronic liver diseases, such as autoimmune and metabolic liver diseases, and to validate the findings with histologically confirmed fibrosis stages.

## Data Availability

The datasets generated and/or analysed during the current study are not publicly available due to legal regulations and data protection requirements in the country of origin but are available from the corresponding author on reasonable request.

## Abbreviations

ALT: Alanine amino transferase
APRI: Aminotransferase-platelet ratio index
AST: Aspartat amino transferase
AUROC: Area under the ROC curve
AUROC: Area under the receiver operating curve
CHC: Chronic Hepatits C infection
CK-18: Cytokeratine 18
COL4-7S: type IV collagen 7s
DAA: Direct acting anti-viral agents
FIB-4: Fibrosis-4 Score
HA: Hyaluronic acid
HCC: Hepatocellular carcinoma
HCV: Hepatitis C Virus
IL-33: Interleukin-33
MASLD: Metabolic dysfunction associated steatotic liver disease
MELD: Model of End-Stage Liver Disease
ROC: Receiver operating characteristic curves
SCCS: Swiss Hepatitis C Cohort Study
sST2: Soluble suppression of tumorigenicity 2
SVR: Sustained virological response
VCTE: Vibration-controlled transient elastography
